# Using Three-Dimensional Computational Modeling to Compare the Geometrical Fitness of Two Kinds of Proximal Femoral Intramedullary Nail for Chinese Femur

**DOI:** 10.1155/2013/978485

**Published:** 2013-02-05

**Authors:** Sheng Zhang, Kairui Zhang, Yimin Wang, Wei Feng, Bowei Wang, Bin Yu

**Affiliations:** ^1^Department of Orthopedics and Traumatology, Nanfang Hospital, Southern Medical University, Guangzhou 510515, China; ^2^Department of Orthopedics, Second Affiliated Hospital to Inner Mongolia Medical University, Hohhot 10020, China

## Abstract

*Objective*. The aim of this study was to use three-dimensional (3D) computational modeling to compare the geometric fitness of these two kinds of proximal femoral intramedullary nails in the Chinese femurs. Computed tomography (CT) scans of a total of 120 normal adult Chinese cadaveric femurs were collected for analysis. With the three-dimensional (3D) computational technology, the anatomical fitness between the nail and bone was quantified according to the impingement incidence, maximum thicknesses and lengths by which the nail was protruding into the cortex in the virtual bone model, respectively, at the proximal, middle, and distal portions of the implant in the femur. The results showed that PFNA-II may fit better for the Chinese proximal femurs than InterTan, and the distal portion of InterTan may perform better than that of PFNA-II; the anatomic fitness of both nails for Chinese patients may not be very satisfactory. As a result, both implants need further modifications to meet the needs of the Chinese population.

## 1. Introduction

Intramedullary fixation device has advantages from the biomechanical point of view and has become increasingly popular in treating unstable trochanteric fractures [1, 2], but serious implant-related complications have been reported, such as femoral shaft fracture [3], cutting out [4], lateral migration of the femoral neck screw [4, 5], and distal locking [6, 7]. In 2003, the AO/ASIF group developed the Proximal Femoral Nail Antirotation (PFNA) to improve the rotational and angular stability with a helical blade which can avoid bone loss that occurs during the drilling and insertion of a standard sliding hip screw [8]. Biomechanical tests also demonstrated a significantly higher cut out resistance in the osteoporotic bone compared to commonly used screw systems [9, 10]. However, lots of intra- and postoperative complications were reported since it was used in Asians, such as pain around the hip and the thigh, femoral shaft fracture, lateral blade migration, and lateral cortex splitting during operation [9, 11, 12]. In response, AO/ASIF developed the PFNA-II, which was developed specifically for Asians. The flat lateral shape at the proximal portion of PFNA-II made it easier to be inserted intraoperatively, and its shortened proximal tip significantly reduced the postoperative hip pain [13, 14], but some Asian patients still complained about thigh pain after operation.

Similarly, a new device has been developed which uses 2 cephalocervical screws in an integrated mechanism allowing linear intraoperative compression and rotational stability of the head/neck fragment (InterTan)  [15]. Biomechanical study shows the InterTan possesses some biomechanical benefits in internal fixation of unstable femoral neck fractures compared with DHS and CS [16]. Meanwhile, clinical outcomes support that the InterTan device appears to be a reliable implant for the treatment of intertrochanteric femoral fracture [15]. But few studies have reported the use of these two implants in Asians. The geometrical fitness between these two implants and Chinese femurs is still uncertain.

The purpose of this study was to compare the geometrical fitness of PFNA-II and InterTan for Chinese femurs and to discuss possible improvements needed for these two implants used in the Chinese population. We used three-dimensional (3D) computational modeling, because it has been well demonstrated that a 3D modeling is more precise than 2D methods by Budge et al. as it is less influenced by body position [17]. 

## 2. Methods

A total of 120 cadaveric specimens of full length femur were selected and CT scanned at Nanfang Hospital, Southern Medical University, Guangzhou, China. The scanning region was set from the top of the great trochanter at 0.75 mm intervals to the distal femoral joint surface. 

The characteristics of the GE 16 row spiral CT scanner were as follows: 120 KV, 300 MA, thickness of 0.75 mm, pitch of 0.75 mm, scan time of 200 ms each (10–12 s total scan time), and window diameter of 360 mm. All CT data were saved in “.dicom” format and imported into reverse engineering software Mimics 10.01 (Materialise, Belgium) for 3D reconstructions. 3D femoral models were established via masks creating, region growing, calculation, and remeshing of 3D objects.

**Figure 1:**
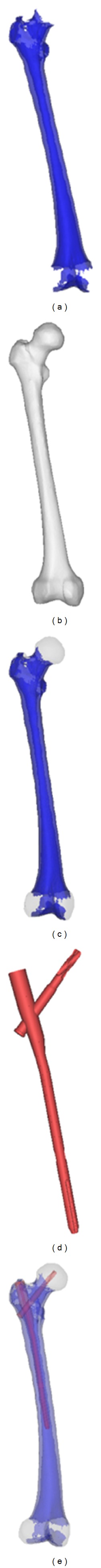
The femur and implant models were reconstructed with software and assembled according to the paper guidelines. (a) The femoral cortex model; (b) the intact femur model. (c) The femoral cortex differentiated from the intact femur model. (d) The PFNA-II model; (e) the implant assembled with the femoral model.

 According to the PFNA-II morphological parameters provided by Synthes and the InterTan morphological parameters provided by Smith & Nephew, 3D models of the two implants were reconstructed with Pro E 4.0 (PTC, MA, USA) software. Then the models of both implants were assembled into the femoral models to compare the geometrical fitness between the implants and the femur (Figure 1).

### 2.1. Position of Implants

The specific location of the whole implant was adjusted until it was in full compliance with the manufacturer's operational guidelines. The antirotation screw was placed in the middle of the femoral head, and the depth of insertion of the intramedullary nail was determined according to the position of the antirotation nail.

### 2.2. Insertion Point

The entry point for the nail was defined according to the manufacturer's guidelines. In the anteroposterior position (AP) view, the entry point was at the vertex of the great trochanter. In the lateral view, the entry point was at the anterior 1/3 of the vertex of the great trochanter. 

In an ideal case, an anatomically shaped nail fits entirely the inside medullary cavity of the bone, indicating that the bone-nail construct stability is optimal, and the axial anatomical alignment of the bone is preserved. Therefore, in this study, the anatomical fitness between nail and bone was assessed by the extent the nail model was impinging or deviating from the medullary cavity of a particular intact (or nonfractured) femoral model. Once an impingement occurred, its incidence, thickness, and length were recorded precisely.

**Figure 2:**
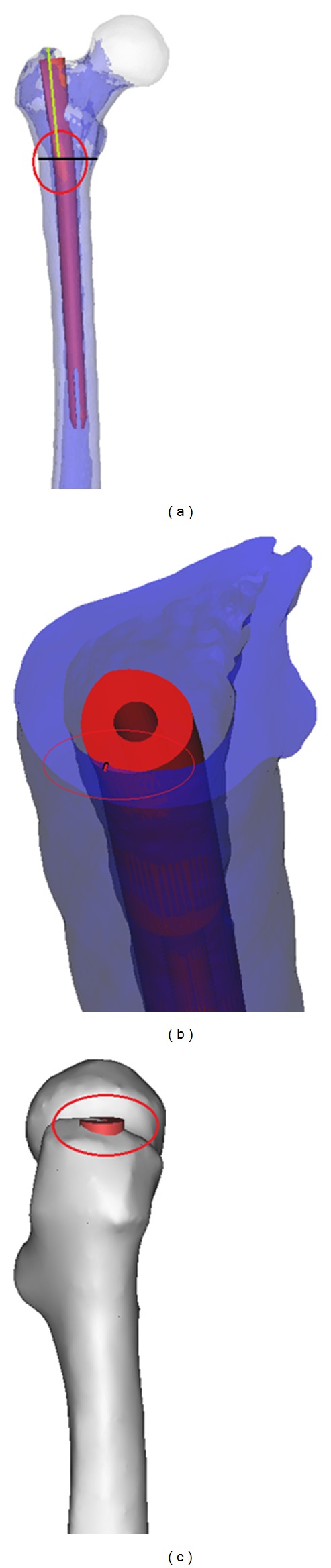
Proximal fitness of the implant with the femoral model. (a) The InterTan assembled with femoral model; the region within the red circle was the region of interest, the model was cut along the black line, and the distance between the top of the great trochanter and the center of the impingement region was depicted in green line. (b) The red line indicated the maximum thickness of the impingement area. (c) Within region the red circle was the proximal end of the implant protruding from the top of the great trochanter.

**Figure 3:**
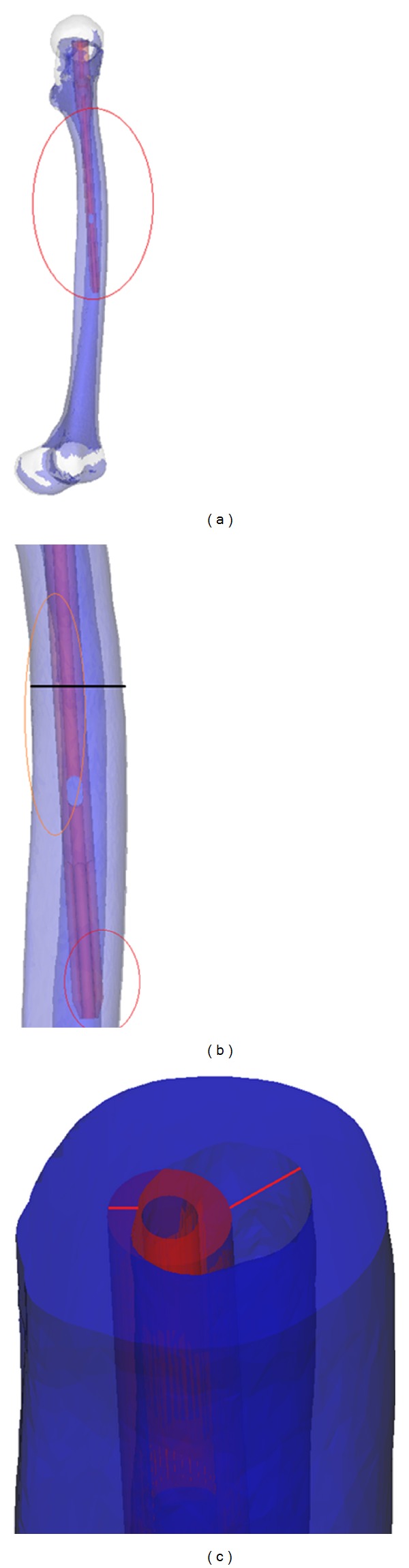
The model was assembled by PFNA-II and femur. (a) The region within the red circle was the region of interest. (b) The impingement area can be easily distinguished within the red circle, and the model was cut along the black line; (c) after the cut of the model, the maximum thickness of the impingement area and the distance from the inner cortex can be measured according to the direction of the red line.

The following geometrical fitness parameters were precisely measured (Figures 2 and 3):

- The greatest thickness an implant impinged on the inner cortex of proximal femoral medullary cavity.
- The average length of an impingement area in proximal femur.
- The distance from the center of an impingement area (proximal) to the top of the great trochanter.
- The length of an implant protruding beyond the top of the great trochanter.
- The length and maximum thickness of a nail impinging the femoral shaft.
- The distance from the center of an impingement area to the top of the great trochanter (middle).
- The maximum distance between the inner cortex and an implant in the middle of femoral shaft.
- The length of an impingement area at the distal part of an implant.

### 2.3. Statistical Analysis

SPSS 16.0 (IBM, USA) was used for statistical analysis. Measurement data are shown in the form of mean ($\overset{-}{X}$) ± SD. The repeated measures test was used to assess the statistical significance of the measurement data. For all tests, *P* < 0.05 was considered significant. Categorical variables were analyzed by the chi-square test or Fisher exact test where appropriate.

## 3. Results

### 3.1. Proximal Fitness

At the proximal part of the nail, the anatomical fitness of these two implants was assessed by criteria (A), (B), (C), and (D). The impingement occurred in 24.2% (*n* = 29) of the InterTan models with an average thickness of 2.53 mm and an average length of 8.61 mm, including 26 cases on the anterior side of the proximal femur and 3 on the lateral. In contrast, the impingement occurred only in 5.8% (*n* = 7) of the PFNA-II models with an average thickness of 1.27 mm and an average length of 10.62 mm, all on the lateral side of the proximal femur. There was a significant difference regarding the impingement incidence between the two models (*P* = 0.003). The average distance from the top of the great trochanter to the center of the impingement area was 57.31 mm in InterTan nails and 62.82 mm in PFNA-II (Table 1). 

The incidence of the implants protruding beyond the top of the great trochanter was 5.8% (*n* = 7) in InterTan nails and 7.5% (*n* = 9) in PFNA-II, the average protruding lenths being 1.05 mm and 1.48 mm, respectively.

### 3.2. Middle Fitness

The fitness at the middle portion of the implant for the femur was analyzed by the values (E), (F) and (G). The impingement occurred in 53.3% (*n* = 64) of the InterTan models and 43.4% (*n* = 52) of the PFNA-II models, and the incidences were comparable (*P* = 0.087). The parameters related to the impingement area were recorded in Table 2. 

The maximum space between the implant and the inner cortex was larger in PFNA-II models than that in InterTan models. The average maximum space was 6.95 mm (SD: 0.68 mm) in the PFNA-II models, much larger than in the InterTan models (4.17 mm ± 0.35 mm) (*P* = 0.035). In the lateral view, for all the models the maximum distance was located at the anterior edge of the nail (Figure 3).

### 3.3. Distal Fitness

At the distal part of the implant, impingement occurred in 31.7% (*n* = 38) of the InterTan models, significantly lower than 52.5% in the PFNA-II models (*n* = 63) (*P* = 0.007). The average length of impingement area was significantly shorter in the InterTan models (8.5 ± 2.13 mm) than in the PFNA-II models (17.6 ± 4.37 mm) (*P* = 0.001). 

## 4. Discussion

Intraoperative and postoperative complications associated with intramedullary nails in the treatment of intertrochanteric fracture can result from imperfect fitness of an internal implant with specific morphology of the human bone. Design of anatomically fitting nails requires morphologic data of the bone that are representative of a specific population. It is well known that fixation devices based on the geometrical proportions of Caucasians do not adequately fit Asian patients, leading to such device-related complications as reported for the Gamma nail [18] and ETN [19]. 

It has been well demonstrated that the PFNA with a bending angle of 6° and a proximal diameter of 17 mm would cause a fracture of the proximal femur during inserting the nail. Otherwise, a larger femoral cannel should be prepared to benefit the nail insert more smoothly [12, 20]. The PFNA-II for Asians was designed to have a bending angle of 5° and a proximal diameter of 16.5 mm. Additionally, the proximal lateral surface was made flat to facilitate insertion and to lower the pressure on the lateral cortex [13]. The present study has demonstrated that the modified nail has a considerably better anatomic fitness for Chinese proximal femur. On the contrary, in this study the InterTan performed worse in the geometrical fitness with Chinese proximal femur, with an impingement incidence of 24.2%. Most impingements occurred on the anterior side of the proximal femur, indicating the anteroposterior diameter of the InterTan may not match well Chinese proximal femurs. Additionally, it is not easy to insert an InterTan nail with a trapezoidal proximal end into a poorly reduced marrow cavity. Consequently, repeated reduction and manipulation may increase both operative and fluoroscopy time and blood loss as well.

Hip and thigh pain [10, 21] seemed to be very common complications in previous reports and could occur in 90.1% of the cases during the follow-up period. One explanation comes to the chronic muscle injury caused by the over-long proximal end of the nail protruding beyond the great trochanter. In this study, both implants matched well the proximal tip of the great trochanter. Clinical results also supported that few patients experienced tenderness following either PFNA-II or InterTan implantation [10, 15]. 

It is known that Chinese women have a shorter femoral neck, smaller femoral neck angles, and increased anterior bowing of the shaft than white American women [18, 19]. It is also acknowledged that the overall stability of the bone-implant construct is important for significant improvements in decreasing postoperative pain and increasing postoperative walking ability. In this study, however, both nails impinged frequently upon the posterior side of the femur at the middle portion of the implant but deviated a lot from the anterior inner cortex from the lateral view. Moreover, both nails did not contact very well the inner cortex at the middle shaft of the femur, due to the Asian characteristic anterior bowing of the femoral shaft. As a result, stress can be concentrated on this region, especially around the locking screw, making the femoral shaft more vulnerable to fracture or leading to long-term thigh pain that cannot be explained. It is suggested that the morphology of the Chinese anterior bowing of the femoral shaft should be taken into consideration when these two nails are used in Asians, especially for those shorter elderly female patients. Furthermore, moderate modification is needed for both implants.

Furthermore, in the present study both implants were biased to the anterior inner cortex at the distal portion from the lateral view. The length of the impingement area was longer in PFNA-II models than in InterTan models. In a similar way, stress may be usually concentrated on this area. It is advised that a shorter PFNA-II should be chosen in treatment of short elderly female patients.

In this study, the impinged areas were mostly located in three regions of the femur. The first region was about 60 mm below the top of the great trochanter and on the anterolateral side of the proximal femur. The second one was located on the posterior side of the femoral shaft, about 155 mm below the top of the great trochanter. The third region was around the distal tip of the nail. In order to design a proximal femoral intramedullary nail fit for Chinese population, the three above regions should also be taken into consideration.

The impressive strength of the present study lies in the simplicity, efficiency, and low cost of the methodology we used to compare the fitness of two implants for a given population, compared with clinical trials. 

In conclusion, although PFNA-II may fit better for the Chinese proximal femurs than InterTan and the distal portion of InterTan may perform better than that of PFNA-II, the anatomic fitness of both nails for Chinese patients may not be very satisfactory. Further clinical results are needed to test the findings of the present 3D computational modeling. In addition, multicenter CT data are also needed to build a database of Chinese bones for 3D modeling which will serve as the basis necessary for the research and development of orthopedic devices for the Chinese population.

## Figures and Tables

**Table 1:** The average fitness value of impingement on the proximal portion of the implant (mm).

	InterTan (*n* = 29)	PFNA-II (*n* = 7)	*P* value
Thickness	2.53 ± 0.58	1.27 ± 0.15	0.017
Length	8.61 ± 1.84	10.62 ± 2.26	0.183
Distance from the top of the great trochanter	57.31 ± 5.42	62.82 ± 5.87	0.213

**Table 2:** The average fitness value on the middle and distal portion of the implant (mm).

	InterTan (*n* = 64)	PFNA-II (*n* = 52)	*P* value
Impingement thickness on the middle shaft of the femur	2.11 ± 0.36	1.62 ± 0.38	0.073
Impingement length on the middle shaft of the femur	60.62 ± 8.37	58.48 ± 7.38	0.361
Distance from the top of the great trochanter to the central of the impingement area	154.43 ± 7.72	148.74 ± 8.87	0.652
Distance between the nail and the inner cortex in the lateral view	4.17 ± 0.35 (*n* = 120)	6.95 ± 0.68 (*n* = 120)	0.035
